# Free triiodothyronine predicts the risk of developing diabetic kidney disease

**DOI:** 10.1186/s12882-023-03349-1

**Published:** 2023-10-11

**Authors:** Weihong Li, Zhi Yang, Shengjian Li, Shanshan Jiang, Wan Hu, Zhenying Wan, Ping Tu, Peng Duan

**Affiliations:** Department of Endocrinology and Metabolism, Nanchang People’s Hospital (The Third Hospital of Nanchang), Jiangxi, China

**Keywords:** Free triiodothyronine, Diabetic kidney disease

## Abstract

**Background:**

Low levels of Free Triiodothyronine (FT3) are associated with poor survival in chronic kidney disease, and the aim of this study was to further assess the relationship between changes in FT3 levels and renal damage in patients with type 2 diabetes based on glomerular and tubular markers.

**Methods:**

We retrospectively studied 452 type 2 diabetic patients, measured glomerular damage markers (UACR, eGFR) and tubular damage markers (NAG/Cr,β2-MG), analyzed the relationship between FT3 and renal damage by logistic regression models, and plotted restrictive cubic splines.

**Results:**

41.6% of subjects had diabetic kidney disease (DKD), and the prevalence of DKD decreased progressively with increasing FT3 levels in the third quartile. Spearman correlation analysis showed that FT3 was negatively associated with UACR, NAG/Cr and β2-MG, while eGFR was positively associated with FT3. Multifactorial analysis, after adjusting for relevant confounders, revealed that compared with the lowest quartile of FT3, the highest quartile reduced the risk of developing urinary albumin (OR = 0.499,95% CI:0.289–0.856), moderate to severe impairment of glomerular filtration rate (OR = 0.106,95% CI:0.032–0.354), renal tubular marker β2 -MG positive (OR = 0.516,95% CI:0.299 to 0.883) and the risk of DKD occurrence (OR = 0.450,95% CI:0.260 to 0.774). In the sample model, FT3 levels below 4.39 pmol/L were associated with an increased risk of glomerular tubule injury and DKD occurrence.

**Conclusions:**

FT3 is closely associated with glomerular tubular injury and is a protective factor. As FT3 levels (< 4.39 pmol/L) decrease, the risk of developing DKD becomes higher, and FT3 can be used as an independent predictor of developing DKD.

## Introduction

Diabetes mellitus is the largest disease in the endocrine field today and is widely prevalent worldwide. Long-term hyperglycemia can involve various organ systems, leading to various tissue, vascular and neurological dysfunctions, among which renal damage is the most common. In recent years, it has been found that the renal damage caused by diabetes is not only limited to the glomerulu, but also the renal tubule play a key role in the early and progressive stages of DKD, and are an important “driver” for the development of DKD [1]. In other words, glomerular and tubule damage is closely related to DKD.

The thyroid, as the largest endocrine gland in the body, is essential for the regulation of energy homeostasis and metabolic rate [2]. It has been shown that the thyroid interacts with the kidney. Thyroid hormone (TH) can directly affect renal growth and development, glomerular filtration rate, renal hemodynamics, and sodium and water homeostasis [3, 4]; the kidney is likewise involved in TH physiology, not only as an organ of TH metabolism and elimination, but also as a target organ for certain effects of iodothyronine [5, 6]. Previous studies have shown a close relationship between thyroid hormones and DKD, Diabetic patients with high thyroid-stimulating hormone (TSH) levels and low FT3 levels are more likely to develop DKD [7], and Subclinical hypothyroidism has been shown to be an independent risk factor for DKD progression [8, 9].

The aim of this study was to further evaluate the relationship between changes in FT3 levels and renal damage in type 2 diabetic patients based on glomerular and tubular markers.

## Materials and methods

### Study Population

A total of 486 patients with type 2 diabetes hospitalized in the endocrinology department of the Third Hospital of Nanchang from 2018 to 2020 were enrolled in this study. The inclusion criteria were patients aged 18 years and older with type 2 diabetes. The following exclusion criteria were considered: other types of diabetes mellitus; those taking drugs affecting thyroid hormone secretion; those with serious damage to the liver, kidney, heart and other organs;those with urinary tract infections, acute infections and those taking drugs affecting urine protein. Thirty-four with dislodged biochemical index information were excluded, and 452 were finally included in the analysis. Ethical approval for this study protocol was obtained from the committee of the Third Hospital of Nanchang.

### Clinical and Laboratory Examination

Relevant information was collected by questionnaire, including demographics, subject characteristics, disease drug history, and lifestyle. Height and weight were measured using an ultrasound instrument (Omron HNH-318, Japan), and body mass index (BMI) was calculated by dividing weight (kg) by height (m^2^). Blood pressure was measured using an electronic sphygmomanometer (Omron HEM-907, Japan), and subjects were instructed to sit relaxed and still for 5 min, keeping the balloon at the same level as the right atrium, and measurements were taken three times, each at 1 min intervals, and the mean value was taken.

Blood samples were collected after patients fasted overnight (at least 8 h). Fasting plasma glucose (FPG), serum creatinine (SCr), serum uric acid (SUA), serum total cholesterol (TC), triglycerides (TG), high-density lipoprotein cholesterol (HDL-C) and low-density lipoprotein cholesterol (LDL-C) were measured by an automated analyzer (Roche, Basel, Switzerland). HbA1c was measured by high-performance liquid chromatography (Bio-Rad D-10, Berkeley, USA). Serum FT3 was measured by chemiluminescent immunoassay (ADVRI2400, Siemens, Germany).

The first early morning urine sample was collected from the subjects. Urinary albumin (immunological turbidimetric method), urinary creatinine (picric acid method), urinary NAG (p-nitrophenol colorimetric and picric acid methods) and urinary β2-MG (immunoturbidimetric method) levels were measured by Siemens ADVRI 2400, Germany. Urinary albumin/creatinine ratio (UACR) and N-acetyl-β-D-glucosaminidase/creatinine ratio (NAG/Cr) were calculated. Estimated glomerular filtration rate (eGFR) was calculated using the equation of the Modification of Diet in Renal Disease: eGFR (mL/min/1.73m2) = 186 × (Scr/88.4) ^− 1.154^ × (age) ^− 0.203^ (*0.742 if female). β2-MG and NAG/Cr reference ranges were ≤ 0.3 mg/L and < 2.4 U/mmol.Cr, respectively.

### Statistical analysis

Data were analyzed using the software SPSS 25.0 and RStudio Statistical descriptions of the count data were expressed as rates (%), and the χ² test was used for comparison between groups. Normally distributed measures were described by mean ± standard deviation, and differences between groups were analyzed by one-way ANOVA. Non-normally distributed ones were expressed as median (interquartile range), and the Kruskal-Wallis H test was used for comparison of differences between groups.Spearman correlation analysis was performed to observe the correlation between FT3 and variables related to kidney injury. Binary logistic regression models were used to analyze the variables associated with kidney injury. We also used restricted cubic splines with four knots at the 5th, 35th, 65th, and 95th centiles to flexibly model the association of FT3 with glomerular damage, tubular damage, and the occurrence of DKD.

## Results

### Clinical characteristics of the patients

FT3 is divided into Q1(≤ 4.11), Q2(4.12–4.63), and Q3(4.64+) groups by tertile。The gender, age, duration of diabetes, smoking, drinking, FPG, HbA1c, BUN, SCr, UACR, eGFR, NAG/Cr and β2-MG were different between the three groups (P < 0.05)( Table 1).

**Table 1:** Comparison of clinical characteristics between FT3 groups

Variables	Q1 (n = 152)	Q2 (n = 153)	Q3 (n = 147)	Total(n = 452)	P-value
Gender(male/female)	60/92	76/77	103/44	239/213	< 0.001
Age(years)	67(57,75)	62(56.5,70)	59(52,65)	62(55,70)	< 0.001
Duration of diabetes(years)	10(4,15)	7(2,11)	5(2,10)	7(3,11)	< 0.001
Smoking(%)	36(8.0%)	47(10.4%)	59(13.1%)	142(31.4%)	0.019
Drinking(%)	6(1.3%)	13(2.9%)	28(6.2%)	47(10.4%)	< 0.001
Hypertension(%)	92(20.4%)	86(19.0%)	74(16.4%)	252(55.8%)	0.206
DKD(%)	90(59.2%)	50(32.7%)	48(32.7%)	188(41.6%)	< 0.001
SBP(mmHg)	132.00(122.00,146.00)	134.00(122.00,143.00)	134.00(124.00,146.00)	133.00(122.50,145.00)	0.807
DBP(mmHg)	80.00(70.00,82.00)	80.00(74.50,86.00)	80.00(76.00,88.00)	80.00(72.50,85.00)	0.124
BMI(kg/m^2^)	24.89(22.23,26.75)	24.71(22.83,27.00)	24.80(22.97,27.29)	24.84(22.53,27.00)	0.304
FPG(mmol/L)	9.08(6.61,12.70)	7.98(6.19,10.72)	7.97(6.69,10.04)	8.33(6.57,11.08)	0.047
HbA1c(%)	9.20(7.30,11.75)	8.70(6.80,10.70)	8.10(6.80,9.70)	8.60(7.00,10.68)	0.001
BUN(mmol/L)	5.84(4.35,7.85)	5.36(4.31,6.24)	5.10(4.28,6.15)	5.37(4.31,6.70)	0.002
SCr(umol/L)	78.50(59.00,107.00)	66.00(53.00,79.50)	66.00(56.00,81.00)	69.00(57.00,88.00)	< 0.001
SUA(umol/L)	289.00(230.00,389.00)	277.00(233.50,343.00)	289.00(244.00,355.00)	286.50(236.00,355.75)	0.397
TG(mmol/L)	4.44(3.65,5.35)	4.44(3.79,5.31)	4.38(3.84,5.01)	4.43(3.75,5.26)	0.795
TC(mmol/L)	1.35(1.02,2.02)	1.43(1.00,2.25)	1.56(1.05,2.44)	1.44(1.03,2.21)	0.704
HDL-C(mmol/L)	1.23(1.05,1.45)	1.21(1.07,1.41)	1.18(1.03,1.39)	1.21(1.05,1.42)	0.396
LDL-C(mmol/L)	2.62(1.91,3.24)	2.59(2.09,3.29)	2.56(2.13,3.23)	2.59(2.06,3.25)	0.714
UACR(mg/g)	36.95(14.74,182.60)	18.09(10.60,45.62)	17.00(9.20,49.37)	23.05(11.31,84.33)	< 0.001
eGFR(ml/min/1.73m^2^)	97.51 ± 44.65	124.43 ± 40.35	129.80 ± 37.13	117.12 ± 43.15	0.019
NAG/Cr(U/mmol.Cr)	1.48(0.91,2.30)	0.89(0.59,1.38)	0.91(0.55,1.53)	1.05(0.64,1.65)	< 0.001
β2-MG(mg/L)	0.22(0.04,1.41)	0.08(0.03,0.26)	0.10(0.04,0.34)	0.10(0.04,0.47)	< 0.001

### Results of the renal function

Table 2 shows the results of eGFR and albuminuria. 73.5% of the subjects had normal or elevated eGFR, 17.3% had a mild decrease, 7.1% had a moderate decrease, 2.0% had a severe decrease, and 0.2% were in renal failure. Regarding urinary albumin excretion, 59.1% of subjects had normal albuminuria, 26.5% had microalbuminuria, and 14.4% had massive albuminuria. The risk of DKD progression was assessed by combining these two renal function indicators according to the recommendations of the Kidney Disease Improvement Global Prognosis Organization (KDIGO) and was found to be low risk in 58.4%, moderate risk in 24.1%, high risk in 11.3% and very high risk in 6.2%.

**Table 2:** Analysis of the proportions of subjects according to the categories of eGFR and UACR

eGFR (ml/min/1.73m^2^)	Albuminuria staging						
	A1 (UACR < 30 mg/g)		A2 (UACR30~300mg/g)		A3 (UACR > 300 mg/g)		Total n(%)
G1 (≥ 90)	226 (50.0%)		77 (17.0%)		29 (6.4%)		332 (73.5%)
G2 (60~89)	38 (8.4%)		29 (6.4%)		11 (2.4%)		78 (17.3%)
G3a (45~59)	3 (0.7%)		11 (2.4%)		8 (1.8%)		22 (4.9%)
G3b (30~44)	0 (0.0%)		2 (0.4%)		8 (1.8%)		10 (2.2%)
G4 (15~29)	0 (0.0%)		1 (0.2%)		8 (1.8%)		9 (2.0%)
G5 (< 15)	0 (0.0%)		0 (0.0%)		1 (0.2%)		1 (0.2%)
Total n(%)	267 (59.1%)		120 (26.5%)		65 (14.4%)		452 (100.0%)

### FT3 levels in different degrees of glomerular and tubular injury

**Fig. 1:**
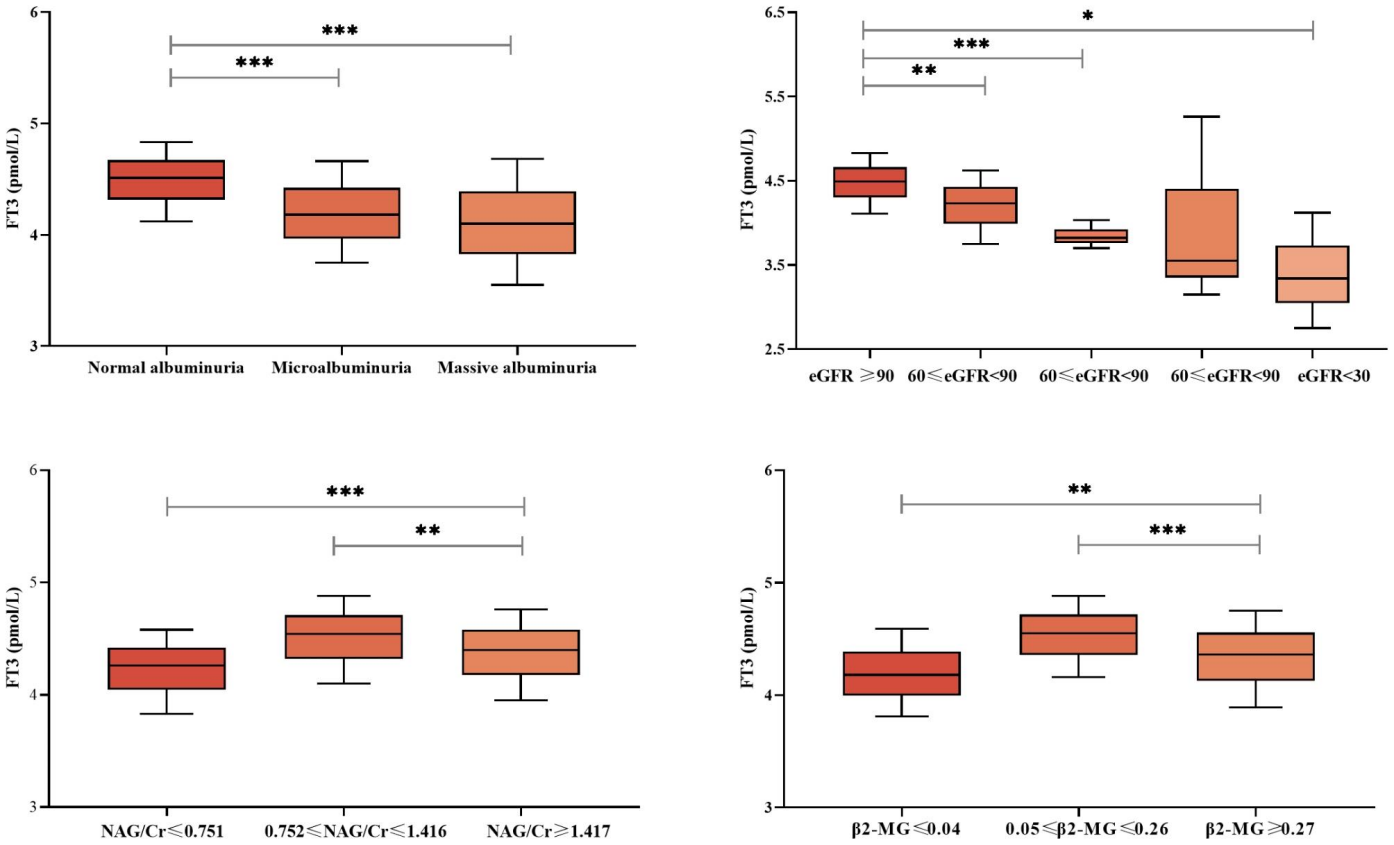
FT3 levels in different degrees of glomerular and tubular injury

Glomerular indices (eGFR, UACR) were grouped by stage and tubular indices (NAG/Cr, β2-MG) were grouped by tertile, and the levels of FT3 were compared between the groups. The results revealed that the comparison of FT3 between groups was statistically different (P < 0.05) (Fig. 1).

### Correlation analysis of FT3 with glomerular and tubular indices

Spearman correlation analysis revealed that FT3 was negatively correlated with UACR, NAG/Cr and β2-MG (P < 0.05). Meanwhile, eGFR was positively correlated with FT3 (P < 0.05)(Table 3).

**Table 3:** Correlation of FT3 with glomerular and tubular indices

Parameters correlate	r value	P value
UACR	-0.267	< 0.001
eGFR	0.325	< 0.001
NAG/Cr	-0.287	< 0.001
β2-MG	-0.155	0.001

### Relationship of FT3 with tubular glomerular injury and diabetic kidney disease

Binary logistic analysis was performed with the presence of albuminuria, the presence of decreased eGFR, the presence of abnormal renal tubular markers (NAG/Cr and β2-MG) and the occurrence of DKD as dependent variables and FT3 as independent variable, respectively. After adjusting for age, BMI, duration of diabetes, history of hypertension, uric acid, HbA1c, and dyslipidemia, FT3 levels were found to be a protective factor. Compared with the lowest quartile of FT3, the highest quartile reduced the risk of developing urinary albumin (OR = 0.499,95% CI:0.289 to 0.856), moderate to severe impairment of glomerular filtration rate (OR = 0.106,95% CI:0.032 to 0.354), positive renal tubular marker β2-MG (OR = 0.516, 95% CI:0.299 to 0.883) and the risk of DKD occurrence (OR = 0.450,95% CI:0.260 to 0.774) (Table 4).

**Table 4:** Regression analysis of FT3 and glomerular and tubular damage and DKD

Characteristics	Groups	Model 1 OR(95%CI)	P value	Model 2 OR(95%CI)	P value
UACR ≥ 30	Q1	1		1	
	Q2	0.363(0.226~0.576)	< 0.001	0.417(0.248~0.695)	< 0.001
	Q3	0.362(0.225~0.578)	< 0.001	0.499(0.289~0.856)	0.012
eGFR < 60	Q1	1		1	
	Q2	0.093(0.027~0.242)	< 0.001	0.106(0.034~0.333)	< 0.001
	Q3	0.097(0.028~0.252)	< 0.001	0.106(0.032~0.354)	< 0.001
NAG/Cr ≥ 2.4	Q1	1		1	
	Q2	0.234(0.106~0.475)	< 0.001	0.296(0.131~0.620)	0.002
	Q3	0.324(0.159~0.628)	0.001	0.540(0.249~1.127)	0.107
β2-MG>0.3	Q1	1		1	
	Q2	0.306(0.184~0.500)	< 0.001	0.353(0.207~0.593)	< 0.001
	Q3	0.374(0.227~0.607)	< 0.001	0.516(0.299~0.883)	0.016
DKD	Q1	1		1	
	Q2	0.334(0.208~0.531)	< 0.001	0.377(0.223~0.630)	< 0.001
	Q3	0.334(0.207~0.533)	< 0.001	0.450(0.260~0.774)	0.004

Model 1: unadjusted.

Model 2:adjusted for, age, BMI, duration of diabetes, history of hypertension,uric acid, HbA1c,dyslipidemia.

**Fig. 2:**
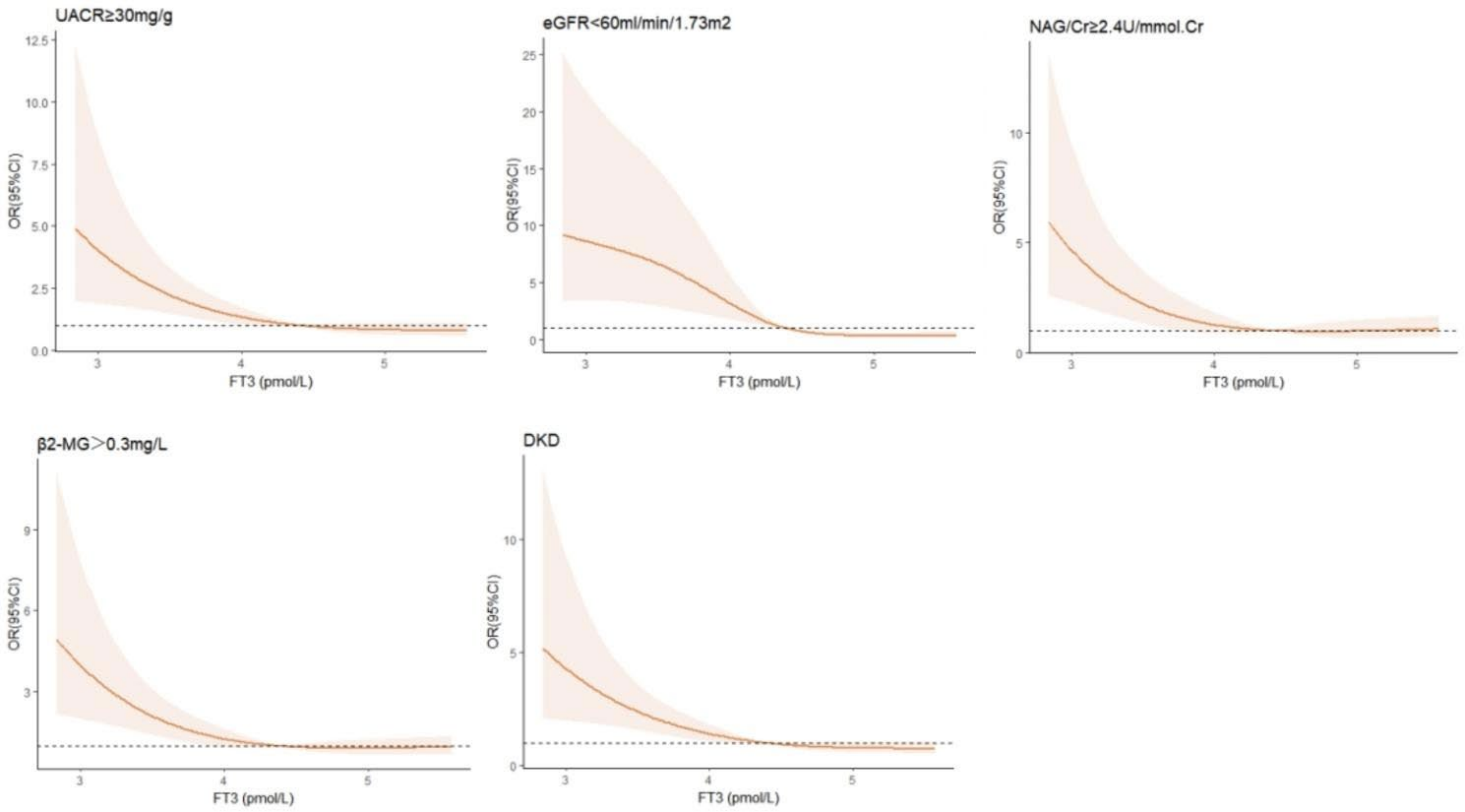
Association of predicted FT3 with tubular glomerular injury and diabetic kidney disease

In Fig. 2, we used restricted cubic splines to flexibly model and visualize the relation of predicted FT3 with glomerular injury, tubular injury, and the occurrence of DKD. The risk of both tubular glomerular injury and DKD development were increased rapidly (P for non-linearity < 0.05) until the predicted FT3 reached 4.39 pmol/L, and then remained relatively flat.

## Discussion

In diabetic kidney disease, the traditional view centered on glomerular alterations has been extended to include tubulointerstitial, immune response and inflammation [10]. Clinical studies have shown that in nearly 2/3 of patients with DKD, there is varying degrees of tubular damage that occurs in the early stages of DKD and may play a key role in the progression of kidney disease [11]. Biomarkers of proximal tubular injury have been shown to be associated with DKD progression and are independent of traditional biomarkers of glomerular injury [12]. These clinical and pathological data strongly suggest that tubular injury plays a key role in the development of DKD and may precede and interact with functional glomerular changes. Thus, glomerular and tubular injury are equally important in the progression of DKD.

FT3 is usually considered the most active thyroid hormone, and when renal function decreases, FT3 levels are reduced in up to 75% of patients [13, 14]. Our study found that the prevalence of DKD gradually decreases as the tripletile of FT3 levels increases. Zou [15] et al. found a negative correlation between FT3 levels and the prevalence of DKD even in patients with T2DM with normal thyroid function. Several cohort and cross-sectional studies found [7, 16–19] that FT3 levels were significantly correlated with UACR and eGFR levels. Our findings are consistent with the above, and we found not only a negative correlation between FT3 levels and UACR and a positive correlation with eGFR, but also a negative correlation with renal tubular markers (NAG/Cr, β2-MG). These studies suggest that reduced FT3 has been the most common impairment in patients with kidney disease.Schultheiss [20] et al. found that higher FT3 levels reduced the risk of all-cause mortality and composite renal endpoints. In our study, FT3 was likewise found to have a protective effect after adjusting for factors related to glomerular and tubular injury. Then we used restrictive cubic spline curves to predict the relationship between FT3 and the risk of glomerular tubular injury and the development of DKD and found that the critical FT3 value was 4.39 pmol/L, and that as FT3 levels (< 4.39 pmol/L) decreased, the risk of DKD increased. These results all suggest that FT3 levels are independently associated with kidney disease and can be used as an independent predictor of the development of DKD.

Several possible mechanisms could explain the link between FT3 and DKD.First, endothelial dysfunction and podocyte lesions. Serum FT3 levels have been shown to be closely associated with endothelial dysfunction in patients with CKD [21], and animal studies have also confirmed that T3 can affect endothelial function by directly or indirectly acting on vascular smooth muscle cells and causing their diastole [22]. In addition, high T3 promoted podocyte re-differentiation and reduced hypertrophy thereby improving renal structure [23]. Second, deacetylase 1 (SIRT1) activity decreased. Studies have shown that SIRT1 is overexpressed in both podocytes and renal tubular cells and attenuates proteinuria and renal injury. The natural metabolite of triiodothyronine (T3) in the deiodination pathway, 3,5-diiodothyronine (T2), prevented a significant decrease in renal SIRT1 protein expression and activity in diabetic rats, thereby protecting the kidney [24].Third, hyperglycemia.Datas suggest that T3 could prevent progressive kidney injury by improving insulin signaling [25–27]. Fourth, Inflammation.In patients with chronic kidney disease, an independent negative correlation between inflammatory cytokines (TNF-α, IL-6 and CRP) and FT3 was found [28, 29]. Interestingly, impaired renal function is a state of high oxidative stress, inflammation and malnutrition that favors low T3 levels [30, 31]. From a renal perspective, the kidney is the primary organ that takes up the thyroid hormone thyroxine (T4) and converts it to the active form triiodothyronine. Renal injury can inhibit the conversion of T4 to T3, resulting in lower serum free T3 concentrations [32].

We are the first to combine glomerular and tubular markers to further assess the relationship between changes in FT3 levels and renal damage in patients with type 2 diabetes. However, the present study also has some limitations. First, this was a cross-sectional study lacking long-term follow-up, and further prospective and longitudinal studies are needed to confirm this. Second, we selected only 2 markers of renal tubular injury based on references [33, 34], which may not be fully representative of renal tubular injury. Third, we did not assess reverse Triiodothyronine (rT3) in the population, and hypotriiodothyronine (T3) syndrome may be present in diabetes.

In conclusion, this study shows that FT3 levels are closely associated with glomerular tubule damage and are a protective factor, and that as FT3 levels (< 4.39 pmol/L) decrease, the risk of developing DKD increases, and FT3 can be used as an independent predictor of developing DKD.

## Data Availability

The data that support the findings of this study are included in the article. Further inquiries can be directed to the corresponding author.

## Abbreviations

FT3: Free Triiodothyronine
DKD: diabetic kidney disease
TH: Thyroid hormone
BMI: body mass index
FPG: Fasting plasma glucose
SCr: serum creatinine
SUA: serum uric acid
TC: serum total cholesterol
TG: triglycerides
HDL-C: high-density lipoprotein cholesterol
LDL-C: low-density lipoprotein cholesterol
UACR: Urine albumin-to-creatinine ratio
NAG/Cr: N-acetyl-β-D-glucosaminidase/creatinine ratio
eGFR: Estimated glomerular fltration rate
β2-MG: β2-microglobulin
CKD: Chronic kidney disease

## References

[1] Yu SM, Bonventre JV (2018). Acute kidney Injury and Progression of Diabetic kidney disease. Adv Chronic Kidney Dis.

[2] Basu G, Mohapatra A (2012). Interactions between thyroid disorders and kidney disease. Indian J Endocrinol Metab.

[3] Meuwese CL, Carrero JJ (2013). Chronic kidney disease and hypothalamic-pituitary axis dysfunction: the chicken or the egg?. Arch Med Res.

[4] Iglesias P, Díez JJ (2009). Thyroid dysfunction and kidney disease. Eur J Endocrinol.

[5] Jusufovic S, Hodzic E (2011). Functional thyroid Disorders are more common in patients on chronic Hemodialysis compared with the General Population. Mater Sociomed.

[6] Kaptein EM (1996). Thyroid hormone metabolism and thyroid diseases in chronic renal failure. Endocr Rev.

[7] Wei L, Bai Y, Zhang Y (2022). Thyroid function and age-related decline in kidney function in older chinese adults: a cross-sectional study. BMC Geriatr.

[8] El-Eshmawy MM, Abd El-Hafez HA, El Shabrawy WO, Abdel Aal IA (2013). Subclinical hypothyroidism is independently associated with microalbuminuria in a cohort of prediabetic egyptian adults. Diabetes Metab J.

[9] Yasuda T, Kaneto H, Kuroda A (2011). Subclinical hypothyroidism is independently associated with albuminuria in people with type 2 diabetes. Diabetes Res Clin Pract.

[10] Alicic RZ, Rooney MT, Tuttle KR (2017). Diabetic kidney disease: Challenges, Progress, and possibilities. Clin J Am Soc Nephrol.

[11] Bagby SP (2007). Diabetic nephropathy and proximal tubule ROS: challenging our glomerulocentricity. Kidney Int.

[12] Yao L, Liang X, Qiao Y, Chen B, Wang P, Liu Z (2022). Mitochondrial dysfunction in diabetic tubulopathy. Metabolism.

[13] Song SH, Kwak IS, Lee DW, Kang YH, Seong EY, Park JS (2009). The prevalence of low triiodothyronine according to the stage of chronic kidney disease in subjects with a normal thyroid-stimulating hormone. Nephrol Dial Transplant.

[14] Cheng SY, Leonard JL, Davis PJ (2010). Molecular aspects of thyroid hormone actions. Endocr Rev.

[15] Zou J, Tian F, Zhang Y (2018). Association between thyroid hormone levels and Diabetic kidney disease in Euthyroid patients with type 2 diabetes. Sci Rep.

[16] Peters J, Roumeliotis S, Mertens PR, Liakopoulos V (2021). Thyroid hormone status in patients with impaired kidney function. Int Urol Nephrol.

[17] Zhang Y, Chang Y, Ryu S (2014). Thyroid hormone levels and incident chronic kidney disease in euthyroid individuals: the Kangbuk Samsung Health Study. Int J Epidemiol.

[18] Rai S, Kumar JA (2013). Thyroid function in type 2 diabetes mellitus and in diabetic nephropathy. J Clin Diagn Res.

[19] Zhou Y, Ye L, Wang T (2014). Free triiodothyronine concentrations are inversely associated with microalbuminuria. Int J Endocrinol.

[20] Schultheiss UT, Steinbrenner I, Nauck M (2020). Thyroid function, renal events and mortality in chronic kidney disease patients: the german chronic kidney Disease study. Clin Kidney J.

[21] Yilmaz MI, Sonmez A, Karaman M (2011). Low triiodothyronine alters flow-mediated vasodilatation in advanced nondiabetic kidney disease. Am J Nephrol.

[22] Ojamaa K, Klemperer JD, Klein I (1996). Acute effects of thyroid hormone on vascular smooth muscle. Thyroid.

[23] Benedetti V, Lavecchia AM, Locatelli M (2019). Alteration of thyroid hormone signaling triggers the diabetes-induced pathological growth, remodeling, and dedifferentiation of podocytes. JCI Insight.

[24] Shang G, Gao P, Zhao Z (2013). 3,5-Diiodo-l-thyronine ameliorates diabetic nephropathy in streptozotocin-induced diabetic rats. Biochim Biophys Acta.

[25] Al-Kafaji G, Malik AN (2010). Hyperglycemia induces elevated expression of thyroid hormone binding protein in vivo in kidney and heart and in vitro in mesangial cells. Biochem Biophys Res Commun.

[26] Ortega E, Koska J, Pannacciulli N, Bunt JC, Krakoff J (2008). Free triiodothyronine plasma concentrations are positively associated with insulin secretion in euthyroid individuals. Eur J Endocrinol.

[27] Lin Y, Sun Z (2011). Thyroid hormone ameliorates diabetic nephropathy in a mouse model of type II diabetes. J Endocrinol.

[28] Zoccali C, Tripepi G, Cutrupi S, Pizzini P, Mallamaci F (2005). Low triiodothyronine: a new facet of inflammation in end-stage renal disease. J Am Soc Nephrol.

[29] Abozenah H, Shoeb S, Sabry A, Ismail H (2008). Relation between thyroid hormone concentration and serum levels of interleukin-6 and interleukin-10 in patients with nonthyroidal illness including chronic kidney disease. Iran J Kidney Dis.

[30] Warner MH, Beckett GJ (2010). Mechanisms behind the non-thyroidal illness syndrome: an update. J Endocrinol.

[31] Carrero JJ, Stenvinkel P, Cuppari L (2013). Etiology of the protein-energy wasting syndrome in chronic kidney disease: a consensus statement from the International Society of Renal Nutrition and Metabolism (ISRNM). J Ren Nutr.

[32] Bianco AC, Dumitrescu A, Gereben B (2019). Paradigms of dynamic control of thyroid hormone signaling. Endocr Rev.

[33] Asare-Anane H, Twum F, Kwaku Ofori E, Torgbor EL, Amanquah SD, Osafo C (2016). Urinary lysosomal enzyme activities and Albuminuria in Ghanaian patients with type 2 diabetes Mellitus. Dis Markers.

[34] Zeng X, Hossain D, Bostwick DG, Herrera GA, Zhang PL (2014). Urinary β2-Microglobulin is a good Indicator of Proximal Tubule Injury: a correlative study with renal biopsies. J Biomark.

